# Quantifying RNA Editing in Deep Transcriptome Datasets

**DOI:** 10.3389/fgene.2020.00194

**Published:** 2020-03-06

**Authors:** Claudio Lo Giudice, Domenico Alessandro Silvestris, Shalom Hillel Roth, Eli Eisenberg, Graziano Pesole, Angela Gallo, Ernesto Picardi

**Affiliations:** ^1^Institute of Biomembranes, Bioenergetics and Molecular Biotechnologies, National Research Council, Bari, Italy; ^2^RNA Editing Lab, Oncohaematology Department, IRCCS Ospedale Pediatrico “Bambino Gesù,” Rome, Italy; ^3^The Mina and Everard Goodman Faculty of Life Sciences, Bar-Ilan University, Ramat Gan, Israel; ^4^School of Physics and Astronomy, Raymond and Beverly Sackler Faculty of Exact Sciences, Tel Aviv University, Tel Aviv, Israel; ^5^Sagol School of Neuroscience, Tel Aviv University, Tel Aviv, Israel; ^6^Department of Biosciences, Biotechnology and Biopharmaceutics, University of Bari, Bari, Italy; ^7^National Institute of Biostructures and Biosystems, Rome, Italy

**Keywords:** RNA editing, transcriptome, RNAseq, deep sequencing, *Alu* editing index

## Abstract

Massive transcriptome sequencing through the RNAseq technology has enabled quantitative transcriptome-wide investigation of co-/post-transcriptional mechanisms such as alternative splicing and RNA editing. The latter is abundant in human transcriptomes in which million adenosines are deaminated into inosines by the ADAR enzymes. RNA editing modulates the innate immune response and its deregulation has been associated with different human diseases including autoimmune and inflammatory pathologies, neurodegenerative and psychiatric disorders, and tumors. Accurate profiling of RNA editing using deep transcriptome data is still a challenge, and the results depend strongly on processing and alignment steps taken. Accurate calling of the inosinome repertoire, however, is required to reliably quantify RNA editing and, in turn, investigate its biological and functional role across multiple samples. Using real RNAseq data, we demonstrate the impact of different bioinformatics steps on RNA editing detection and describe the main metrics to quantify its level of activity.

## Introduction

Eukaryotic organisms exhibit quite complex and dynamic transcriptomes whose regulation is essential for all cellular processes and for maintaining the homeostatic state (Mele et al., 2015). The complexity and dynamicity of transcriptomes depends on highly controlled and modulated post-transcriptional mechanisms such as alternative splicing and RNA modifications (Pan et al., 2008; Meyer and Jaffrey, 2014; Roundtree et al., 2017). The latter are now emerging as key players in promoting transcriptome diversity and fine tuning gene expression (Helm and Motorin, 2017; Roundtree et al., 2017). Transient and non-transient RNA modifications belong to the epitranscriptome world (Schwartz, 2016; Tajaddod et al., 2016; Boccaletto et al., 2018). Non-transient modifications occurring in a variety of RNA molecules and organisms through base insertions/deletions or substitutions are referred to as RNA editing changes (Gott and Emeson, 2000). In mammals, the most common RNA editing event involves the deamination of adenosine (A) into inosine (I), carried out by members of the ADAR family of enzymes acting on double stranded RNA (dsRNA) (Nishikura, 2016; Eisenberg and Levanon, 2018).

Deep transcriptome sequencing, through the RNAseq technology, has greatly promoted identification of RNA editing events at genomic scale, revealing the extent of A-to-I editing in humans, with more than 4.6 million modification sites identified so far. The majority of RNA editing modifications (>95%) resides in *Alu* repetitive elements that are widespread in human genes (accounting for around 10% of the human genome) (Levanon et al., 2004). Transcripts harboring two such elements with inverted orientations may fold to form dsRNA structures targeted by ADARs. In contrast, only a minute fraction of RNA editing events occurs in protein-coding genes and can lead to recoding, i.e., non-synonymous substitutions that generate novel protein isoforms. Recoding sites are enriched in neural tissues and over-represented in transcripts encoding proteins linked to the nervous system function (Rosenthal and Seeburg, 2012).

Accumulating evidence indicates that A-to-I RNA editing in mammals modulates the innate immune response (Mannion et al., 2014) and its deregulation has been observed in various human diseases including autoimmune and inflammatory tissue injury (Gallo and Locatelli, 2012; Roth et al., 2018; Shallev et al., 2018; Vlachogiannis et al., 2019), neurodegenerative and psychiatric disorders (Khermesh et al., 2016; Breen et al., 2019; Tran et al., 2019), and tumors (Gallo, 2013; Han et al., 2015; Paz-Yaacov et al., 2015; Silvestris et al., 2019).

An important property of RNA editing is that its levels vary across different tissues and cell types. Both the edited and unedited versions of transcripts co-exist in the same tissue or cell and the ratio between the unedited and edited variants is regulated by a variety of factors depending on tissue type or developmental stage. Consequently, quantifying RNA editing, detecting levels of edited variants or measuring the overall editing activity, are crucial for investigating its functional involvement and biological role.

A variety of bioinformatics programs and workflows have been released to profile RNA editing in deep transcriptome datasets (Picardi et al., 2015a; Diroma et al., 2019; Lo Giudice et al., 2020). Although based on different algorithms, all of them predict RNA editing candidates mitigating biases mainly due to sequencing errors, mapping errors, and genomic SNPs (Diroma et al., 2019). Hereafter, we describe a number of important metrics to quantify RNA editing in RNAseq experiments, enabling comparative analysis of whole inosinomes across multiple samples. Using real RNAseq data, we elaborate on different bioinformatics steps that have an impact on the profiling of RNA editing. These include pre-processing of raw reads or the specific strategy for alignment to the genome. As of to date no single computational methodology guarantees detection of all real editing events occurring in a sample, and the specific procedures for RNA editing detection and quantification in a given RNAseq dataset should be carefully selected, bearing in mind that the same procedure should be applied to all samples of a study to allow comparison of the results.

## Methods

### RNAseq Samples, Pre-processing, and Alignment

RNAseq data from four tissues and 10 “body sites” (Table 1 and Supplementary Table S1) were downloaded from Genotype-Tissue Expression (GTEx) Project through the dbGAP accession phs000424. Raw data were initially inspected using FASTQC and reads were trimmed using FASTP. Then, high quality reads were aligned onto the human genome (hg19 assembly from UCSC) using STAR v.2.5.2b (Dobin et al., 2013), providing a list of known gene annotations from GENCODE (Derrien et al., 2012). In addition, human cerebellum reads (accession SRR607967) were aligned to the human genome (hg19 and hg38 primary assemblies) using BWA v.0.7.17 (Li and Durbin, 2009) and HISAT2 v.2.1.0 (Kim et al., 2015) with known splice sites and exons from GENCODE.

**TABLE 1:** Summary table of experiments used.

Tissue	Body site	N. samples
Artery	Aorta	14
Artery	Tibial	14
Brain	Amygdala	13
Brain	Cerebellum	12
Brain	Frontal cortex	13
Brain	Hippocampus	11
Brain	Hypothalamus	14
Brain	Spinal cord	10
Lung	Lung	9
Muscle	Skeletal	13

### RNA Editing Detection

A list of *de novo* RNA editing candidates per sample was generated using REDItools, following the filtering procedure described in Picardi et al. (2015b) and Lo Giudice et al. (2020). Aligned reads from run SRR607967 were also analyzed by JACUSA (Piechotta et al., 2017) using common basic filters. Hyper-edited reads were identified using the computational procedure described by (Porath et al., 2014).

### RNA Editing Quantification

The overall RNA editing level *per* sample was calculated using a custom python script, taking as input a list of positions inferred by REDItools. The same program was also used to quantify RNA editing levels at known positions, downloaded from REDIportal database (including more than 4.5 million events in humans). The robustness of the overall editing metric over the number of RNA editing positions was tested selecting randomly growing numbers of positions from the REDIportal collection and calculating the overall editing *per* each sampling and “body site.” Then, we measured the Pearson correlation between the overall editing calculated *per* each group of positions and the same metric detected using the whole database collection, by means of a custom script (pearsonr function from scipy python module).

Recoding index was also calculated using a custom python script working on REDItools tables. We considered as recoding sites all 1585 editing positions in REDIportal that are marked as non-synonymous in all three gene annotations available in the database (RefSeq, UCSC, and GENCODE). *Alu* editing index (AEI) was calculated using the methodology by Roth et al. (2019).

### Differential RNA Editing

Differential RNA editing at REDIportal recoding sites was identified using the non-parametric Mann–Whitney (MW) *U*-test. Recoding sites were collected *per* each artery tibial and cerebellum sample from REDItools tables. The comparison was carried out by a custom python script taking into account sites covered by at least 10 RNAseq reads in at least 50% of the samples *per* group. *p*-values were corrected for multiple testing using the Benjamini–Hochberg method.

Software, command lines, and scripts used in this work are available at the following GitHub repository https://github.com/BioinfoUNIBA/QEdit.

## Results and Discussion

### Pre-processing and Alignment of RNAseq Experiments

Profiling RNA editing in whole transcriptome data is yet a challenging task, due to sequencing errors, read-mapping errors, genome-encoded polymorphisms (SNPs), somatic mutations, and spontaneous RNA chemical changes. SNPs and somatic mutations may be partly filtered out using genomic reads from matched whole genome sequencing (WGS) or whole exome sequencing (WXS) experiments, as well as tables of known SNPs from public databases. Alignment and sequencing errors may be partly removed using stringent filters of read and base quality. All of these aforementioned issues require careful design and tuning of computational pipelines to detect RNA editing candidates, as each step or procedure or software can affect the yield and quality of predictions.

**FIGURE 1:**
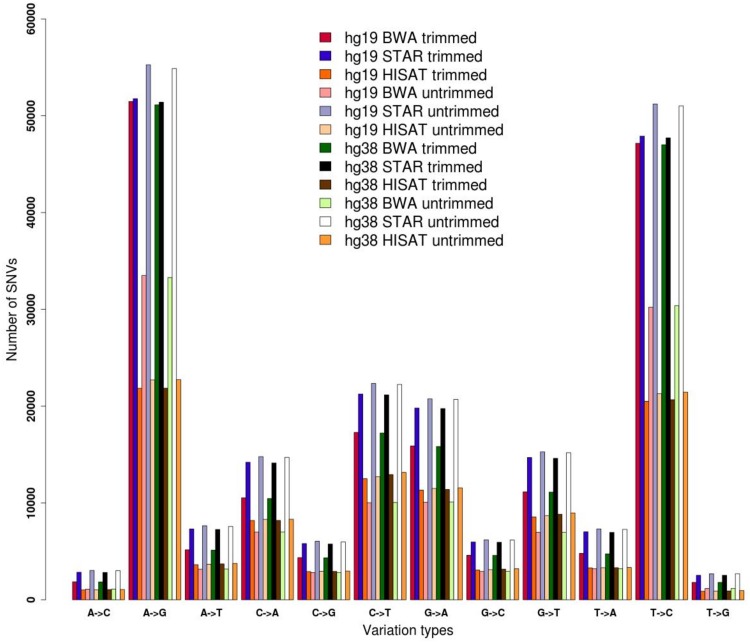
Distribution of single nucleotide variants detected by REDItools on trimmed and untrimmed reads (from accession SRR607967) aligned by means of BWA, STAR, and HISAT2 onto hg19 and hg38 human genome assemblies.

Here we demonstrate the effects of pre-processing and genome alignment steps on RNA editing calling using a single GTEx RNAseq experiment from human cerebellum (run accession SRR607967). Raw reads were initially inspected using FASTQC and their low quality regions were removed by means of FASTP. Two datasets were generated, the first containing original raw reads and the second including trimmed reads. Both datasets were aligned onto the hg19 and hg38 reference chromosomes of the human genome using three different aligners, BWA designed for unspliced reads and STAR and HISAT2 optimized for handling spliced reads. Resulting multi-alignments were processed with REDItools in order to provide the distribution of single RNA variants according to a common basic filtering scheme. Known SNPs from the WGS of the same individual (run accession SRR2165704) were removed. In all tested cases, we achieved quite similar distributions, in which A-to-G and T-to-C changes (putative editing events on the direct or reverse strand) are over-represented, suggesting enrichment in true RNA editing events (Figure 1). However, the number of detected sites varied depending on the processing steps, suggesting that the trimming procedure as well as the aligner type affect the detection of RNA editing. The three different aligners resulted in different results, reflecting the slightly different algorithms. STAR has returned the highest number of candidates. Surprisingly, HISAT2 yielded the lowest number of variants, even though it is splice-aware and did align the same proportion of reads as STAR (Figure 1 and Supplementary Table S2).

**FIGURE 2:**
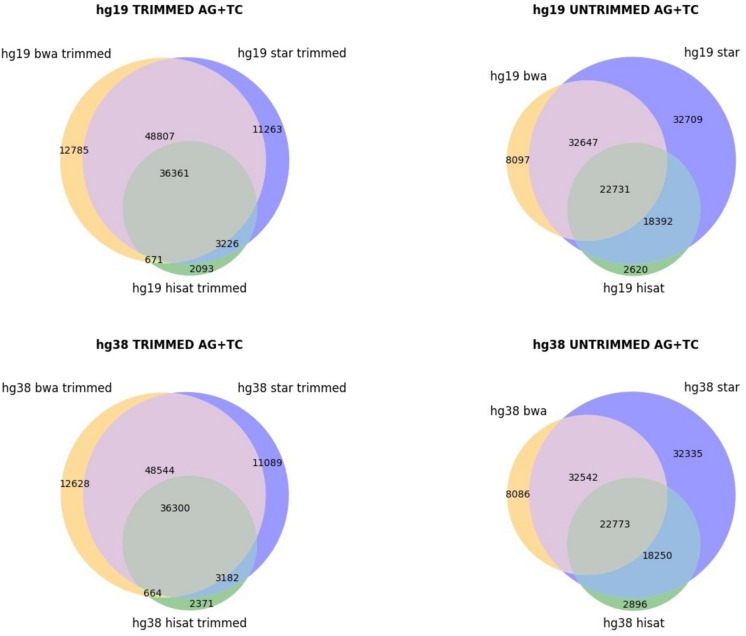
Venn diagrams, showing the AG/TC overlapping positions for BWA, STAR, and HISAT2 aligners. The comparison is made for trimmed and untrimmed reads mapping onto hg19 and hg38 assemblies, respectively.

The genome version used (hg19 and hg38 human genome assemblies) did not make an appreciable difference (Figure 1), but the alignment of raw or trimmed reads did have an aligner-dependent effect (Figure 1). Although deviations in all checked cases do not appear graphically marked, they do influence the final list of candidates (Figure 2). We thus see that simple computational steps or the adoption of specific software can dramatically change the final results and impact commonly used metrics for quantification of global or local RNA editing activity in a sample. Adopting the same computational pipeline to analyze multiple samples or compare results from already published works is highly recommended.

### RNA Editing Detection

Once trimming and alignment steps have been performed, the final list of RNA editing candidates strongly depends on the methodology used to call them. In general, two types of approaches can be pursued, *de novo* or “known”. The former aims to identify all potential RNA editing events of a sample or the hyper-edited regions only without relying on previously known sets of editing positions, while the latter focuses on a restricted number of known changes from literature or well-established databases.

#### *De novo* Approach

Several software packages to detect *de novo* RNA editing events in deep transcriptome data have been released to date. They all suffer from some level of false positives, and each tool requires fine tuning of a variety of parameters that can strongly affect the quality of results and, thus, sensitivity and specificity of predictions (Diroma et al., 2019). The behavior of several RNA editing detection programs has been recently assessed (Diroma et al., 2019). Here we analyze comparatively two *de novo* approaches for RNA editing identification, REDItools (Picardi and Pesole, 2013) and JACUSA (Piechotta et al., 2017), using the same aligned human cerebellum reads. The two methods require traversing multiple alignments of reads through a pileup function. REDItools detect events applying different empirical filters while JACUSA implements a statistical model for variant calling. Both tools were applied to trimmed reads aligned onto the hg19 genome by STAR, followed by common basic filters such as the removal of sites in homopolymeric stretches longer than five residues or falling in the first and last six bases of a read, the exclusion of positions covered by less than 10 reads and showing a phred quality score less than 30.

**FIGURE 3:**
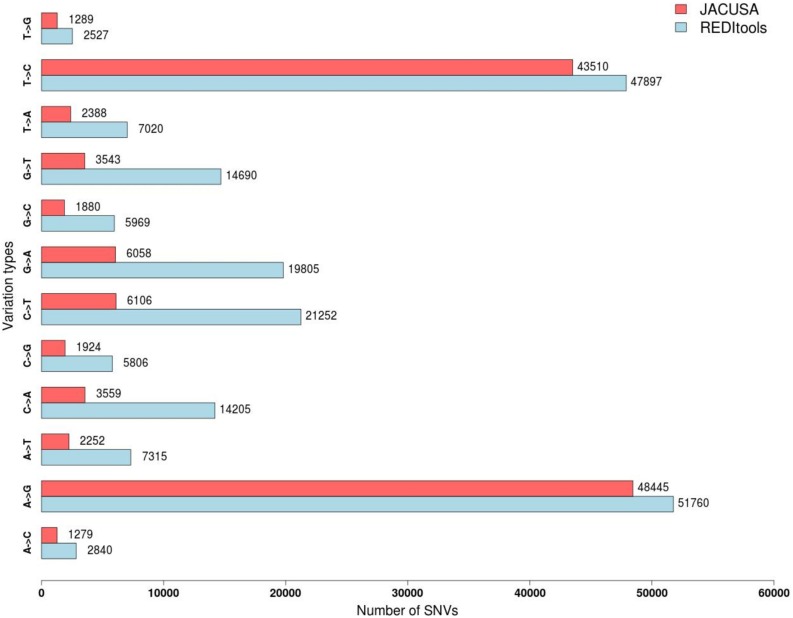
Distribution of single nucleotide variants detected by JACUSA vs REDItools on trimmed reads SRR607967 aligned by STAR on hg19 human genome assembly.

**FIGURE 4:**
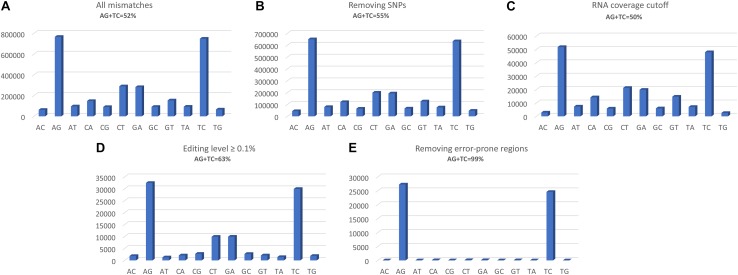
Distributions of RNA variants detected by REDItools obtained following the different filtering steps: **(A)** all mismatches found following mapping, with a phred quality score of at least 30; **(B)** selecting only sites supported by at least 10 WGS reads and removing positions in dbSNP; **(C)** selecting sites covered by at least 10 reads and not falling in homopolymeric stretches longer than five residues or in the first and last six bases of a read; **(D)** selecting sites with an editing frequency of at least 0.1; **(E)** excluding sites in mis-mapped reads (by Blat correction) or near splice sites or in genomic regions containing poorly aligned reads.

The two programs return a similar number of variants, but with different precision. REDItools yielded 99,657 putative editing sites (49.56% of all observed modification sites) while JACUSA predicted 91,955 putative editing sites (75.23% of all observed modification sites) (Figure 3). In this specific example, JACUSA appeared more stringent than REDItools showing a higher signal-to-noise ratio, likely due to its statistical model and further filtering step by a companion R script, the JacusaHelper. This example demonstrates that RNA editing calling tools should be used with care, paying attention in advance to the various combinations of parameters and the experimental features of samples. A good practice is to estimate the false discovery rate comparing the A-to-G fraction (and T-to-C for unstranded reads) with the noise due to other base changes not expected to be edited, and then tune the parameters accordingly. Indeed, multiple filters can greatly improve the quality of final results. For example, to mitigate mapping errors (by Blat re-alignment) and other spurious changes occurring near splice sites or in genomic regions containing poorly aligned reads we applied more stringent filters to REDItools (Lo Giudice et al., 2020). Doing so, the number of variants detected in the same sample dropped down to only 52,400 sites including about 99% (51,888 positions) of potential RNA editing events (A-to-G and T-to-C changes) with a very low estimated false discovery rate, <1%. The effect of the different filtering steps on the distribution of RNA variants is shown in Figure 4. Importantly, the third step (coverage cut-off) results in a sizable drop in the number of excess AG/TC mismatches. While this step is necessary in order to achieve a good signal-to-noise ratio, one should bear in mind that the vast majority of the signal is lost during this step.

Note that in other species, e.g., mice, *Alu* elements are not present and the number of expected RNA editing candidates is much lower compared to humans (Neeman et al., 2006; Ramaswami and Li, 2014). This might require re-tuning the alignment and calling parameters. Furthermore, in case multiple samples from biological replicates are available, these may be used to further improve final results, looking only at putative RNA editing candidates common to all replicates.

#### “Known” Approach

The *de novo* approach generates a list of candidate sites likely to be edited in the specific RNAseq dataset. Sometimes, however, it could be useful to focus on a set of known events in order to better investigate RNA editing dynamics in different experimental contexts. For example, RNA editing could be profiled in known recoding events of neurotransmitter receptors to study its involvement in synaptic function or its deregulation in neurological/psychiatric disorders or cancer (Gallo, 2013; Han et al., 2015; Paz-Yaacov et al., 2015; Khermesh et al., 2016; Silvestris et al., 2019). REDItools package is the most suitable tool for this task (Picardi and Pesole, 2013). Providing a list of genomic positions and a pre-aligned file of RNAseq reads, it recovers the exact site and the corresponding RNA editing level. The “known” approach has been successfully applied also to large scale genomic projects. In the specialized database REDIportal (Picardi et al., 2016), for example, REDItools have been used to interrogate multiple read alignments from 2660 GTEx RNAseq experiments employing a large collection of known RNA editing sites from the ATLAS repository (Picardi et al., 2015b) and DARNED database (Kiran et al., 2013). Another example is The Cancer RNA Editome Atlas (TCEA) (Lin and Chen, 2019), where REDIportal positions (4,656,896) have been explored in more than 11,000 RNAseq data from the TCGA project (Cancer Genome Atlas Research Network et al., 2013).

#### Hyper-Editing

ADAR enzymes are known to have the ability to deaminate clusters of adjacent adenosines leading to hyper-edited RNA molecules (Eisenberg, 2016). Many RNA editing calling programs, however, fail to discover hyper-editing events because of the high number of mismatches *per* read that avoids its correct alignment on the genome (Porath et al., 2014). Heavily edited reads can be detected through a specific computational protocol in which not aligned sequences are rescued and mapped again onto a transformed genome replacing As with Gs (Porath et al., 2014). Since hyper-editing occurs mainly in *Alu* repetitive elements, it could lead to altered AEI values with a trend to underestimate the RNA editing activity *per* sample. As an example, we applied the computational strategy by Porath et al. (2014) to the above cerebellum RNAseq experiment (run accession SRR607967) using 3,490,661 unmapped reads by STAR. The alignment onto the transformed human genome yielded 19,377 reads enriched in A-to-G clusters, corresponding to 124,546 RNA editing changes. Of these, only 3586 were present in the filtered list of candidates by REDItools. Consequently, more than 120,000 A-to-G RNA editing events, missed by REDItools in the previous analysis, have been *de novo* identified in hyper-edited regions. So, events falling in hyper-edited reads should not be excluded *a priori* since they may represent a considerable fraction of sites. Large scale investigations based on TCGA samples have proven that the number of unique editing sites identified in hyper-edited regions follows the same trend as the AEI index calculated excluding hyper-edited reads (Paz-Yaacov et al., 2015). These findings suggest that the expected AEI underestimation does not significantly affect the global RNA editing activity measured at *Alu* level.

### Metrics for RNA Editing Quantification

Once RNA editing has been detected in RNAseq samples, quantification is the next step that allows comparing values across samples and study of the potential role of RNA editing in different experimental conditions, such as its involvement in human disorders. Quantification of RNA editing is also important to identify molecular markers that could be the target for engineered ADAR enzymes or modified CRISPR-Cas systems, according to the recent paradigm of the precision medicine. Quantification of RNA editing has always been a challenging task, especially in the last few years in which deep transcriptome sequencing has enabled large scale investigations. Several metrics have been proposed, some of them take into account the global RNA editing activity (Tan et al., 2017; Roth et al., 2019), while other approaches focus on specific sites only (Khermesh et al., 2016; Silvestris et al., 2019). Below, we illustrate the main metrics using GTEx RNAseq data from four tissues and ten “body sites” (see section “Methods” for further details).

#### Overall Editing Level

**FIGURE 5:**
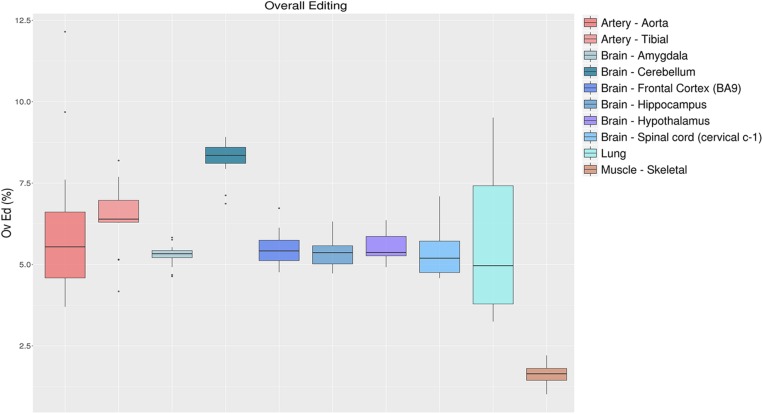
Overall editing levels in 10 selected “body sites” from the GTEx project. Each box plot represents samples from one tissue type. The overall editing level is defined as the percentage of edited nucleotides at all known editing sites. Cerebellum and skeletal muscle emerge, respectively, as the most edited tissue and the less-edited tissue among the analyzed tissues.

To quantify the global RNA editing in a sample, one can average the editing levels measured over the sites detected previously, or by *de novo* methods (Tan et al., 2017). This metric, referred to as the overall editing, is determined as the total number of reads with G at all known editing positions over the number of all reads covering the positions without imposing specific sequencing coverage criteria (Tan et al., 2017). The overall editing depends on the number of known editing sites included in the analysis that have to be the same for all samples analyzed. Using *de novo* editing events for this purpose is not recommended, as the number of detected sites is unevenly distributed across samples and strongly depends on the amount of raw reads input and the bioinformatics procedure (Picardi et al., 2015b; Diroma et al., 2019). Even merging *de novo* candidates from all samples of interest does not remove the coverage bias altogether. Alternatively, one may calculate the overall editing employing known events stored in public databases such as REDIportal (Picardi et al., 2016), RADAR (Ramaswami and Li, 2014), or DARNED (Kiran et al., 2013). To illustrate the behavior of the overall editing index, we calculated this metric in 123 GTEx RNAseq experiments from 10 “body sites” employing REDIportal as it stores the largest public collection of human RNA editing annotations (4,665,677 sites in its last release). As shown in Figure 5, RNA editing appeared reduced in skeletal muscle compared to other tissues, as already observed in previous studies (Picardi et al., 2015b; Tan et al., 2017). On the contrary, cerebellum displayed the highest RNA editing level. These results are consistent with the *Alu* editing level among “body sites” (Roth et al., 2019) with cerebellum emerging as the top tissue carrying the highest editing level, higher that other brain regions including cortex. It has been estimated that there are about 3.6 times as many neurons in the cerebellum as in the cortex (Herculano-Houzel, 2010). Possibly, the higher level in cerebellum is merely a result of a higher fraction of neurons in this tissue, as neurons are highly edited compared to other brain cells (Gal-Mark et al., 2017).

**FIGURE 6:**
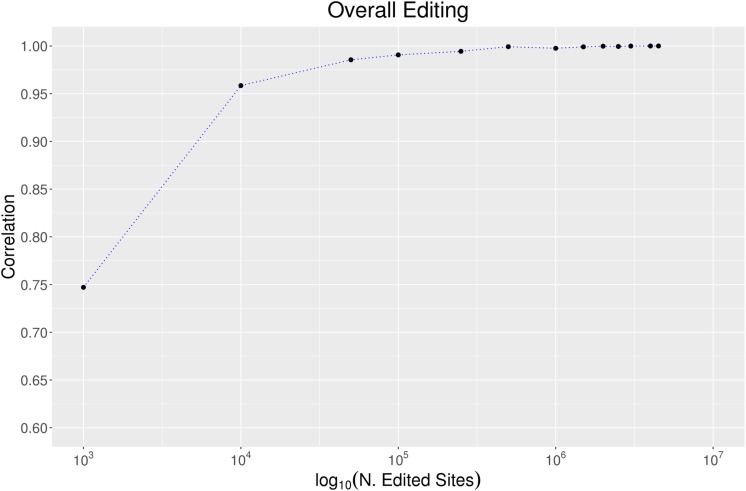
The effect of the number of sites on the overall editing. We calculated the overall editing calculated in all 123 GTEx samples using a growing number of positions randomly selected from REDIportal database. The Pearson correlations between the overall editing measured per each group of positions and the same metric on the entire REDIportal collection are depicted.

To evaluate the effect of the number of RNA editing positions on the robustness of the overall editing metric, we randomly selected growing numbers of positions from the REDIportal collection and calculated the overall editing *per* each sampling and “body site.” Assuming the highest accuracy when all REDIportal positions are used, we measured the correlation between the overall editing calculated *per* each group of positions and the same metric detected using the whole database collection. As reported in Figure 6, 100,000 RNA editing positions are sufficient to obtain a very high correlation (Pearson *R* = 0.99 Pval << 10^–4^) with the entire REDIportal database. Using the RNA editing sites from DARNED (333,215 sites) and RADAR (2,576,459 sites), we obtained a correlation with REDIportal of 95% (Pval << 10^–4^) and 99% (Pval << 10^–4^), respectively.

#### *Alu* Editing Index

**FIGURE 7:**
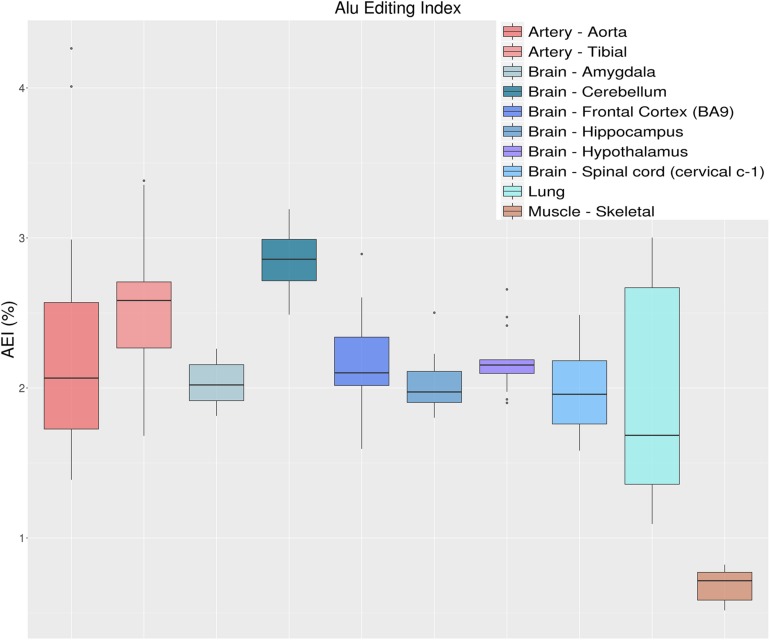
Distributions of *Alu* editing index (AEI) values over 10 selected tissue types from the GTEx project. AEI represents the weighted average editing level across all expressed *Alu* sequences. Distributions are presented as box-plots. AEI clearly recapitulates the same trend as overall editing thus confirming that the sites in *Alu* regions are those that have the greatest impact on the global editing activity.

Another metric to quantify the global RNA editing activity is to calculate the weighted average of editing events occurring in all adenosines within *Alu* elements, defined as the AEI. As mentioned above, the vast majority of editing activity takes place within *Alu* elements, with almost every adenosine in the ADAR-targeted *Alu* repeats being edited to some extent (Bazak et al., 2014a). The AEI is defined to be the ratio (for convenience in percentage) of the number observed A-to-G mismatches to the total coverage of adenosines (both A-A matches and presumed editing events, A-to-G mismatches). It is therefore the weighted average of the measured editing levels weighted by the coverage of each site (Bazak et al., 2014b). The AEI avoids the quantification of editing rates per-sites, while accounting for editing in lowly covered regions. It also frees the user from dependence on public databases that might be continuously changing (or even unavailable for other species). Since the AEI is calculated over millions of positions it is highly robust to the number of input raw reads, and as few as one million input reads already provide a consistent and almost invariable signal (Roth et al., 2019). It is, however, affected by the alignment process (i.e., aligner and read lengths), but preserves the relative rank of each sample. As an example, Figure 7 shows the distribution of AEI values for 123 GTEx samples, calculated as described in Roth et al. (2019). Results indicate a general agreement between the measured AEI and the overall editing index depicted above (Figure 5). It should be noted that this approach is not limited to the human genome. One can use the index for any organism, as long as a large set of highly editable elements (often, SINE elements) is available and the editing is strong enough to result in a sufficiently large signal-to-noise ratio.

#### Recoding Index

Similarly to the overall editing, recoding activity due to RNA editing could be quantified, focusing on editing levels at recoding positions (residing in coding protein genes). For example, one may calculate the weighted average over all known recoding sites, known as the recoding editing index (REI) (Silvestris et al., 2019). This measure is well correlated with ADAR2 expression, at least in normal brain (Silvestris et al., 2019), and may be a good indicator of ADAR2 deaminase activity. Interestingly, REI may be utilized to investigate RNA editing deregulation in different brain regions or neurological disorders (Khermesh et al., 2016) or cancer (Silvestris et al., 2019). REI is simply defined as the number of reads with G at recoding positions over the number of all reads covering the same positions (same as AEI, but for the recoding sites). As in the case for the overall editing, the reliability of REI depends on the number of recoding sites to assay. Indexing over very small numbers, e.g., the 35 recoding sites known to be conserved across the mammalian lineage (Pinto et al., 2014), could lead to biased values and misleading conclusions. The list of recoding sites can be obtained from databases such as REDIportal (Picardi et al., 2016), RADAR (Ramaswami and Li, 2014), or DARNED (Kiran et al., 2013). However, one should bear in mind that the false positive level of recoding sites in these public collections is notoriously high.

**FIGURE 8:**
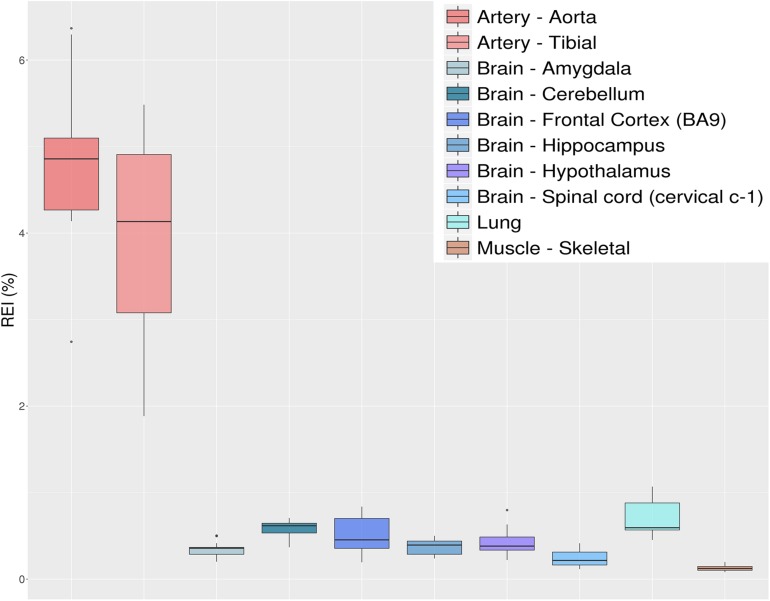
Distributions of recoding editing index (REI) values over 10 selected tissues from the GTEx project reported as box-plots. REI is calculated as the weighted average of editing levels over all known recoding sites from the REDIportal database. Most brain sub-tissues show similar levels of recoding editing. A remarkable exception is represented by the aorta and tibial artery showing a surprisingly high editing level.

**FIGURE 9:**
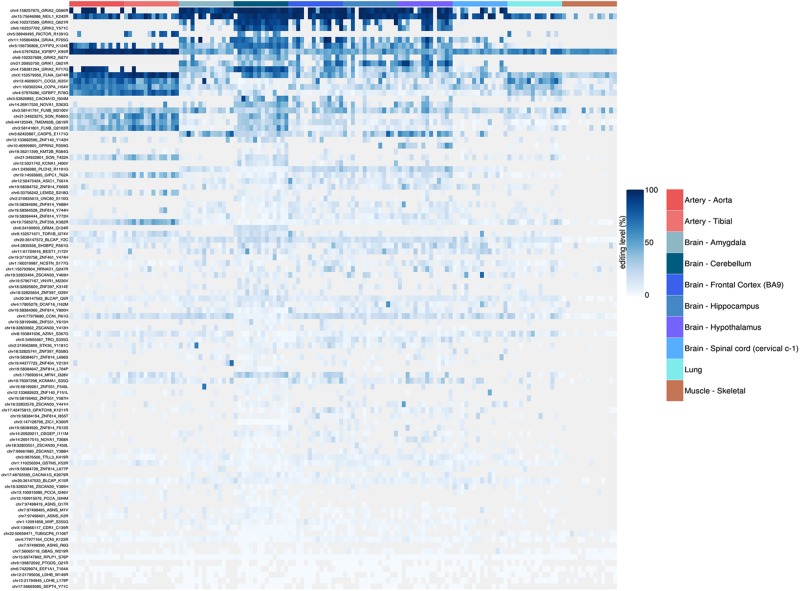
Heatmap representing RNA editing levels at 99 selected recoding events. Body sites are reported in the same order as in the previous box-plots and follow the same color code. The hierarchical clustering (dendrogram not shown) of the recoding sites shows how the artery (both aorta and tibial) are characterized by a very peculiar and specific set of strongly (>90%) edited sites, thus suggesting a possible key functional role of these sites in the vascular system.

Here, we show the REI results using 1585 non-synonymous RNA editing events from REDIportal (see selection criteria in section “Methods”) for the above GTEx RNAseq experiments (Figure 8). Our results, similarly to those by Tan et al. (2017) from the complete GTEx dataset, show a very high recoding activity at arteries compared to other tissues, with lung and brain being at similar levels and skeletal muscle showing the lowest REI levels. Of note, the ADAR2 expression level (as shown by GTEx in Supplementary Figure S1) overlaps well the results shown in Figure 8. So far, many studies, including ours, have underlined the important role played by RNA editing at recoding sites in the central nervous system (CNS). In contrast, the role of A-to-I RNA editing in angiogenesis, artery, endothelium, and vascular disease was only recently explored (Stellos et al., 2016; Jain et al., 2018). While Stellos et al. (2016) have pointed to ADAR1 activity within the 3′ untranslated region (3′ UTR) of cathepsin S mRNA (*CTSS*), Jain et al. (2018) reported that recoding at *FLNA* (Q/R) is an important regulator of vascular contraction and blood pressure. Our data and a previous study (Jain et al., 2018) indicated the presence of some almost fully edited sites in artery, similar to the *GRIA2* Q/R in CNS, and extended the list of important recoding sites in artery that may play a crucial role in vascular physiology and diseases (Figure 9). Indeed, among the top edited genes in arteries, there is the Insulin-like growth factor-binding protein 7 (IGFBP7). IGFBP7 is a secreted protein involved in diverse biological functions, from apoptosis to inhibition/stimulation of growth and angiogenesis (Brahmkhatri et al., 2015). Proteolytic processing of IGFBP7 modulates its biological activity as it can stimulate growth of DLD−1 colon carcinoma cells in synergy with insulin/IGF−I but, if cleaved, IGFBP7 completely abolishes this growth-stimulatory activity (Ahmed et al., 2003). Interestingly, editing of *IGFBP7* transcripts (K/R site) affects the protein’s susceptibility to proteolytic cleavage, thus providing a means for a cell to modulate its multiple activity through A-to-I RNA editing (Godfried Sie et al., 2012).

The REI is a measure of global RNA editing activity at recoding sites. However, one should bear in mind that recoding activity is often unevenly distributed across the different sites. High REI values could mean overall high recoding activity, but might also occur at a few highly expressed and highly edited sites only. In the aforementioned artery samples, for instance, three recoding events in *IGFBP7* and *FLNA* transcripts account for more than 90% of all edited Gs, and for the high value of the REI as compared to other tissues. In case one is interested in the distribution, a common practice is to look at graphical visualizations of editing levels through all sites of interest, using, for example, a heatmap plot (Figure 9).

### Differential RNA Editing

An important question related to the RNA editing profiling is the identification of differentially edited sites. A variety of statistical tests have been proposed so far, but reliable, consistent, and reproducible detection of dysregulated RNA editing is still a major task. The observed A-to-I levels at individual sites depend strongly on the methodology used to call them, sequencing depth and coverage. Events residing in repetitive elements, comprising the majority of A-to-I changes, exhibit low levels (typically lower than 0.01), requiring ultra-high coverage for reliable detection and quantification. A given position could appear edited in some samples but unedited in others (because of limited coverage), a fact that is often ignored in the statistical testing. Sometimes, when the number of samples is sufficiently high, missing editing levels could be imputed using methods based on the principal component analysis (Josse and Husson, 2016), chained equations (Buuren and Groothuis-Oudshoorn, 2011), or random forest (Stekhoven and Bühlmann, 2012).

Finally, the large number of editing sites requires an aggressive multiple-testing correction, and severely limits the statistical power. This leads to an underestimate of the number of differentially edited sites.

**FIGURE 10:**
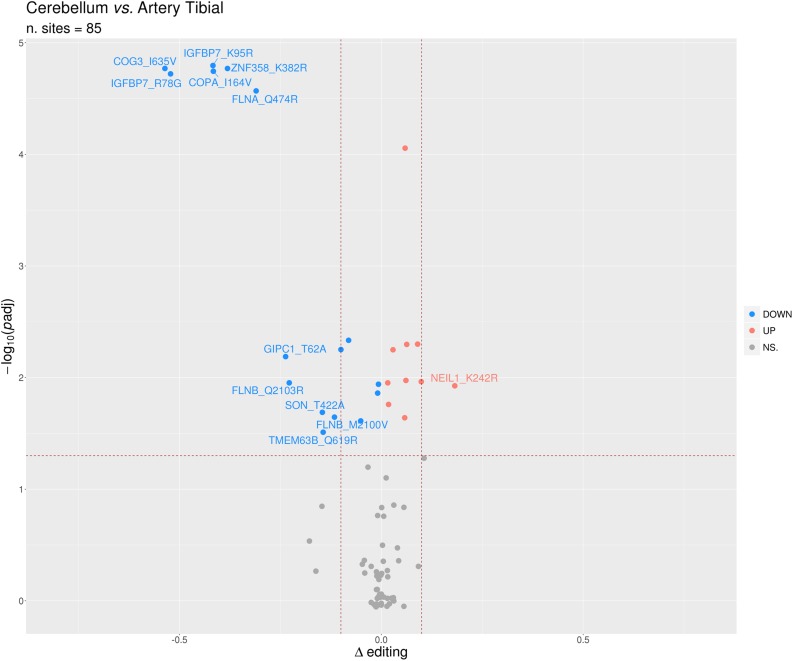
Volcano plot reporting the differentially edited sites between cerebellum and tibial artery. The horizontal dotted line marks a multiple test-corrected level of significance (adjusted padj < 0.05, Mann–Whitney with Benjamini–Hochberg correction). The vertical dotted lines indicate a Delta editing of 0.1 and -0.1. Red, blue, and gray points indicate, respectively, over-edited (UP) sites, under-edited (DOWN) sites, and non-significative sites (NS.).

Identification of differential RNA editing is most relevant at recoding sites, where altered A-to-I levels could lead to different protein isoforms. Editing dysregulation at recoding sites between two groups of samples is often assayed applying the two-tailed MW *U*-test followed by Benjamin–Hochberg multiple test corrections. For example, such an approach was used to identify many recoding sites differentially edited in cancer compared with normal samples (Maas et al., 2001; Paz et al., 2007; Cenci et al., 2008; Chen et al., 2013; Qin et al., 2014; Han et al., 2015; Paz-Yaacov et al., 2015; Hu et al., 2016; Lin and Chen, 2019; Silvestris et al., 2019). Here, we demonstrate this approach by detecting statistically significant differentially recoded sites between 14 artery tibial and 12 cerebellum samples, looking at 1585 non-synonymous REDIportal positions quantified using REDItools. We considered only sites supported by at least 10 RNAseq reads in at least the three samples per group, thus obtaining 85 positions to test for differential RNA editing levels (Figure 10). Of these, 26 sites, residing in 21 target genes, were statistically significant (Table 2). Sixteen positions appeared more edited in artery tibial than cerebellum while 10 appeared more edited in cerebellum than in artery tibial (Table 2). Sites showing higher differences in RNA editing levels belonged to well-characterized target genes such as *COG3* (Han et al., 2015; Peng et al., 2018; Silvestris et al., 2019), *IGFBP7* (Chen et al., 2017), *COPA* (Han et al., 2015; Peng et al., 2018), *FLNA* (Riedmann et al., 2008; Jain et al., 2018), and *ZNF358* (Zhang et al., 2016; Lee et al., 2017). The functional impact of RNA editing at these substrates is mostly unknown.

**TABLE 2:** Statistically significant differential recoding sites.

Chr:position	Gene	AA change	Δ editing	Pval (MW)	Padj (BH)
chr4:57976234*	IGFBP7	K95R	–0.417	0.000015	0.000016
chr13:46090371	COG3	I635V	–0.536	0.000016	0.000017
chr19:7585273	ZNF358	K382R	–0.381	0.000016	0.000017
chr1:160302244*	COPA	I164V	–0.416	0.000017	0.000018
chr4:57976286*	IGFBP7	R78G	–0.522	0.000019	0.000019
chrX:153579950*	FLNA	Q474R	–0.310	0.000027	0.000027
chr4:17805279	DCAF16	I162M	0.059	0.000088	0.000088
chr8:103841636	AZIN1	S367G	–0.081	0.000218	0.004633
chr20:36147572*	BLCAP	Y2C	0.090	0.000295	0.005015
chr20:36147563	BLCAP	Q5R	0.063	0.000178	0.005043
chr4:77979680	CCNI	R61G	–0.100	0.000132	0.005610
chr20:36147533	BLCAP	K15R	0.029	0.000464	0.005634
chr19:14593605	GIPC1	T62A	–0.237	0.000458	0.006488
chr12:133682596	ZNF140	Y142H	0.061	0.000998	0.010604
chr14:26917530	NOVA1	S363G	0.099	0.000128	0.010880
chr3:9876560	TTLL3	K419R	0.016	0.001442	0.011143
chr3:58141801*	FLNB	Q2103R	–0.228	0.001180	0.011144
chr5:156736808	CYFIP2	K124E	–0.007	0.001350	0.011475
chr15:75646086	NEIL1	K242R	0.182	0.001673	0.011850
chr4:77977164	CCNI	K123R	–0.009	0.002107	0.013777
chr1:12091858	MIIP	S355G	0.018	0.002867	0.017407
chr21:34922801*	SON	T422A	–0.146	0.003614	0.020479
chr3:58141791	FLNB	M2100V	–0.116	0.004256	0.022610
chr18:32825609	ZNF397	K314E	0.058	0.004582	0.022910
chr10:79397298	KCNMA1	S35G	–0.051	0.005179	0.024456
chr6:44120349*	TMEM63B	Q619R	–0.144	0.006899	0.030864

Per each position we report the target gene and amino-acid change induced by RNA editing, the difference between mean editing levels of groups, the Mann–Whitney *p*-value, and the adjusted *p*-value by Benjamin–Hochberg. Positive **Δ** values indicate higher editing in cerebellum than artery, while negative Δs are associated to lower editing in cerebellum than artery. Positions marked by * are differentially edited by also the Tran et al. statistical pipeline.

As an alternative to MW *U*-test, deregulated A-to-I editing has been identified using the statistical pipeline proposed by Tran et al. (2019) to detect dysregulated RNA editing in brains of autistic individuals. In this case, differential RNA editing sites are defined as positions having significantly different average editing levels between autistic donors and controls, or observed at significantly different population frequencies (Tran et al., 2019). Editing candidates are ranked by read coverage and the Wilcoxon rank-sum test is used if at least five samples in both control and donor groups have the required depth (Tran et al., 2019). By applying this pipeline to the above data, we found 10 differentially edited sites, eight of them already detected by the MW *U*-test (Table 2).

To date performance of statistical tests for differential RNA editing has never been tested and systematically assessed. Typically, the tests applied ignore the inherent noise introduced by the limited reads’ coverage. Generally, tests assuming a normal distribution of RNA editing levels (such as the *t*-test) should be avoided. Indeed, accumulating evidence from large scale projects indicates that RNA editing levels seem to follow a beta distribution rather than a normal distribution (Picardi et al., 2015b). Further investigations are, in any case, needed to better understand the statistical properties of RNA editing levels.

## Conclusion

RNAseq is currently the technology of choice for large-scale studies of transcriptional and co-/post-transcriptional mechanisms. In the last few years, several computational tools have been developed to profile A-to-I editing in a variety of RNAseq data. Yet, RNA editing prediction is still not a fully solved bioinformatics task. However, noise and biases due to sequencing errors, read-mapping errors, and SNPs can be partly mitigated pre-processing reads and fine tuning program parameters depending on the selected algorithm.

The accurate detection of A-to-I editing is indispensable to systematically quantify RNA editing and facilitate comparative investigations across multiple samples. Similarly, A-to-I quantification metrics should be carefully selected. Indeed, measuring RNA editing activity across samples counting *de novo* detected sites or averaging over *de novo* sites leads to very noisy and confounding results. RNA editing is unevenly distributed across samples and different intrinsic (read quality, coverage, or depth) and extrinsic (mapping tool, read pre-processing, RNA editing calling software) factors affect the *de novo* detection that is far from being exhaustive. Averaging over millions of known sites from public databases can help but it requires estimated RNA editing levels that are dependent on a prefixed coverage cut-off that, in turn, drastically reduces the number of usable sites and leads to unreliable, often irreproducible, measures. The weighted average (or an index) over millions of known sites from public database, named here as the overall editing, is a much better solution. However, using this approach one has to rely on a specific set of sites from a given database, a set that might be continuously being modified. In contrast, the AEI is calculated over all tens of millions of genomic adenosines located within *Alu* sequences and accounts for the editing activity in low covered regions, while avoiding the need to quantify the editing level per-site. An index similar to AEI can be determined for recoding events. However, as the number of recoding sites is much lower, and the current set is known to be very noisy, the REI, while informative in some cases, should be used with care.

Identification of differential RNA editing is an important task. Although many studies have been employing various parametric and non-parametric approaches, further investigations are required. Given the non-normal distribution of RNA editing levels, and the strong (yet, usually ignored) effect of variable coverage, *ad hoc* models may be probably required to better perform this task.

## Data Availability Statement

Publicly available datasets were analyzed in this study. This data can be found here: dbGAP accession phs000424.

## Author Contributions

CL and DS performed main bioinformatics analyses. SR carried out AEI computations. EE and GP supervised the work. AG and EP conceived the study and designed the analyses. EP drafted the manuscript. All authors approved the final version of the manuscript.

## Conflict of Interest

The authors declare that the research was conducted in the absence of any commercial or financial relationships that could be construed as a potential conflict of interest.

## References

[B1] Ahmed S. Yamamoto K. Sato Y. Ogawa T. Herrmann A. Higashi S. (2003). Proteolytic processing of IGFBP-related protein-1 (TAF/angiomodulin/mac25) modulates its biological activity. *Biochem. Biophys. Res. Commun.* 310 612–618. 10.1016/j.bbrc.2003.09.058 14521955

[B2] Bazak L. Haviv A. Barak M. Jacob-Hirsch J. Deng P. Zhang R. (2014a). A-to-I RNA editing occurs at over a hundred million genomic sites, located in a majority of human genes. *Genome Res.* 24 365–376. 10.1101/gr.164749.113 24347612 PMC3941102

[B3] Bazak L. Levanon E. Y. Eisenberg E. (2014b). Genome-wide analysis of Alu editability. *Nucleic Acids Res.* 42 6876–6884. 10.1093/nar/gku414 24829451 PMC4066801

[B4] Boccaletto P. Machnicka M. A. Purta E. Piatkowski P. Baginski B. Wirecki T. K. (2018). MODOMICS: a database of RNA modification pathways. 2017 update. *Nucleic Acids Res.* 46 D303–D307. 10.1093/nar/gkx1030 29106616 PMC5753262

[B5] Brahmkhatri V. P. Prasanna C. Atreya H. S. (2015). Insulin-like growth factor system in cancer: novel targeted therapies. *BioMed. Res. Int.* 2015:538019. 10.1155/2015/538019 25866791 PMC4383470

[B6] Breen M. S. Dobbyn A. Li Q. Roussos P. Hoffman G. E. Stahl E. (2019). Global landscape and genetic regulation of RNA editing in cortical samples from individuals with schizophrenia. *Nat. Neurosci.* 22 1402–1412. 10.1038/s41593-019-0463-7 31455887 PMC6791127

[B7] Buuren S. V. Groothuis-Oudshoorn K. (2011). mice: Multivariate Imputation by Chained Equations in R. *J. Stat. Softw.* 45 1–67. 10.18637/jss.v045.i03

[B8] Cancer Genome Atlas Research Network, Weinstein J. N. Collisson E. A. Mills G. B. Shaw K. R. M. Ozenberger B. A. (2013). The cancer genome atlas pan-cancer analysis project. *Nat. Genet.* 45 1113–1120. 10.1038/ng.2764 24071849 PMC3919969

[B9] Cenci C. Barzotti R. Galeano F. Corbelli S. Rota R. Massimi L. (2008). Down-regulation of RNA editing in pediatric astrocytomas: ADAR2 editing activity inhibits cell migration and proliferation. *J. Biol. Chem.* 283 7251–7260. 10.1074/jbc.M708316200 18178553

[B10] Chen L. Li Y. Lin C. H. Chan T. H. Chow R. K. Song Y. (2013). Recoding RNA editing of AZIN1 predisposes to hepatocellular carcinoma. *Nat. Med.* 19 209–216. 10.1038/nm.3043 23291631 PMC3783260

[B11] Chen Y.-B. Liao X.-Y. Zhang J.-B. Wang F. Qin H.-D. Zhang L.. (2017). ADAR2 functions as a tumor suppressor via editing IGFBP7 in esophageal squamous cell carcinoma. *Int. J. Oncol.* 50, 622–630. 10.3892/ijo.2016.3823 28035363 PMC6903889

[B12] Derrien T. Johnson R. Bussotti G. Tanzer A. Djebali S. Tilgner H. (2012). The GENCODE v7 catalog of human long noncoding RNAs: analysis of their gene structure, evolution, and expression. *Genome Res.* 22 1775–1789. 10.1101/gr.132159.111 22955988 PMC3431493

[B13] Diroma M. A. Ciaccia L. Pesole G. Picardi E. (2019). Elucidating the editome: bioinformatics approaches for RNA editing detection. *Brief. Bioinform.* 20 436–447. 10.1093/bib/bbx129 29040360

[B14] Dobin A. Davis C. A. Schlesinger F. Drenkow J. Zaleski C. Jha S. (2013). STAR: ultrafast universal RNA-seq aligner. *Bioinformatics* 29 15–21. 10.1093/bioinformatics/bts635 23104886 PMC3530905

[B15] Eisenberg E. (2016). Proteome diversification by genomic parasites. *Genome Biol.* 17:17. 10.1186/s13059-016-0875-6 26832153 PMC4736476

[B16] Eisenberg E. Levanon E. Y. (2018). A-to-I RNA editing – immune protector and transcriptome diversifier. *Nat. Rev. Genet.* 19 473–490. 10.1038/s41576-018-0006-1 29692414

[B17] Gallo A. (2013). RNA editing enters the limelight in cancer. *Nat. Med.* 19 130–131. 10.1038/nm.3072 23389604

[B18] Gallo A. Locatelli F. (2012). ADARs: allies or enemies? The importance of A-to-I RNA editing in human disease: from cancer to HIV-1. *Biol. Rev. Camb. Philos. Soc.* 87 95–110. 10.1111/j.1469-185X.2011.00186.x 21682836

[B19] Gal-Mark N. Shallev L. Sweetat S. Barak M. Billy Li J. Levanon E. Y. (2017). Abnormalities in A-to-I RNA editing patterns in CNS injuries correlate with dynamic changes in cell type composition. *Sci. Rep.* 7:43421. 10.1038/srep43421 28266523 PMC5339895

[B20] Godfried Sie C. Hesler S. Maas S. Kuchka M. (2012). IGFBP7’s susceptibility to proteolysis is altered by A-to-I RNA editing of its transcript. *FEBS Lett.* 586 2313–2317. 10.1016/j.febslet.2012.06.037 22750143

[B21] Gott J. M. Emeson R. B. (2000). Functions and mechanisms of RNA editing. *Annu. Rev. Genet.* 34 499–531. 10.1146/annurev.genet.34.1.499 11092837

[B22] Han L. Diao L. Yu S. Xu X. Li J. Zhang R. (2015). The genomic landscape and clinical relevance of A-to-I RNA editing in human cancers. *Cancer Cell* 28 515–528. 10.1016/j.ccell.2015.08.013 26439496 PMC4605878

[B23] Helm M. Motorin Y. (2017). Detecting RNA modifications in the epitranscriptome: predict and validate. *Nat. Rev. Genet.* 18 275–291. 10.1038/nrg.2016.169 28216634

[B24] Herculano-Houzel S. (2010). Coordinated scaling of cortical and cerebellar numbers of neurons. *Front. Neuroanat.* 4:12. 10.3389/fnana.2010.00012 20300467 PMC2839851

[B25] Hu J. Xu J. Pang L. Zhao H. Li F. Deng Y. (2016). Systematically characterizing dysfunctional long intergenic non-coding RNAs in multiple brain regions of major psychosis. *Oncotarget* 7 71087–71098. 10.18632/oncotarget.12122 27661005 PMC5342065

[B26] Jain M. Mann T. D. Stulić M. Rao S. P. Kirsch A. Pullirsch D. (2018). RNA editing of Filamin A pre-mRNA regulates vascular contraction and diastolic blood pressure. *EMBO J.* 37:e94813. 10.15252/embj.201694813 30087110 PMC6166124

[B27] Josse J. Husson F. (2016). missMDA: a package for handling missing values in multivariate data analysis. *J. Stat. Softw.* 70 1–31. 10.18637/jss.v070.i01

[B28] Khermesh K. D’Erchia A. M. Barak M. Annese A. Wachtel C. Levanon E. Y. (2016). Reduced levels of protein recoding by A-to-I RNA editing in Alzheimer’s disease. *RNA* 22 290–302. 10.1261/rna.054627.115 26655226 PMC4712678

[B29] Kim D. Langmead B. Salzberg S. L. (2015). HISAT: a fast spliced aligner with low memory requirements. *Nat. Methods* 12 357–360. 10.1038/nmeth.3317 25751142 PMC4655817

[B30] Kiran A. M. O’Mahony J. J. Sanjeev K. Baranov P. V. (2013). Darned in 2013: inclusion of model organisms and linking with Wikipedia. *Nucleic Acids Res.* 41 D258–D261. 10.1093/nar/gks961 23074185 PMC3531090

[B31] Lee S. H. Kim H. P. Kang J. K. Song S. H. Han S. W. Kim T. Y. (2017). Identification of diverse adenosine-to-inosine RNA editing subtypes in colorectal cancer. *Cancer Res. Treat.* 49 1077–1087. 10.4143/crt.2016.301 28161934 PMC5654148

[B32] Levanon E. Y. Eisenberg E. Yelin R. Nemzer S. Hallegger M. Shemesh R. (2004). Systematic identification of abundant A-to-I editing sites in the human transcriptome. *Nat. Biotechnol.* 22 1001–1005. 10.1038/nbt996 15258596

[B33] Li H. Durbin R. (2009). Fast and accurate short read alignment with Burrows-Wheeler transform. *Bioinformatics* 25 1754–1760. 10.1093/bioinformatics/btp324 19451168 PMC2705234

[B34] Lin C.-H. Chen S. C.-C. (2019). The cancer editome atlas: a resource for exploratory analysis of the adenosine-to-inosine RNA editome in cancer. *Cancer Res.* 79 3001–3006. 10.1158/0008-5472.CAN-18-3501 31015229

[B35] Lo Giudice C. Tangaro M. A. Pesole G. Picardi E. (2020). Investigating RNA editing in deep transcriptome datasets with REDItools and REDIportal. *Nat. Protoc.* 10.1038/s41596-019-0279-7 31996844

[B36] Maas S. Patt S. Schrey M. Rich A. (2001). Underediting of glutamate receptor GluR-B mRNA in malignant gliomas. *Proc. Natl. Acad. Sci. U.S.A.* 98 14687–14692. 10.1073/pnas.251531398 11717408 PMC64742

[B37] Mannion N. M. Greenwood S. M. Young R. Cox S. Brindle J. Read D. (2014). The RNA-editing enzyme ADAR1 controls innate immune responses to RNA. *Cell Rep.* 9 1482–1494. 10.1016/j.celrep.2014.10.041 25456137 PMC4542304

[B38] Mele M. Ferreira P. G. Reverter F. DeLuca D. S. Monlong J. Sammeth M. (2015). The human transcriptome across tissues and individuals. *Science* 348 660–665. 10.1126/science.aaa0355 25954002 PMC4547472

[B39] Meyer K. D. Jaffrey S. R. (2014). The dynamic epitranscriptome: N6-methyladenosine and gene expression control. *Nat. Rev. Mol. Cell Biol.* 15 313–326. 10.1038/nrm3785 24713629 PMC4393108

[B40] Neeman Y. Levanon E. Y. Jantsch M. F. Eisenberg E. (2006). RNA editing level in the mouse is determined by the genomic repeat repertoire. *RNA* 12 1802–1809. 10.1261/rna.165106 16940548 PMC1581974

[B41] Nishikura K. (2016). A-to-I editing of coding and non-coding RNAs by ADARs. *Nat. Rev. Mol. Cell Biol.* 17 83–96. 10.1038/nrm.2015.4 26648264 PMC4824625

[B42] Pan Q. Shai O. Lee L. J. Frey B. J. Blencowe B. J. (2008). Deep surveying of alternative splicing complexity in the human transcriptome by high-throughput sequencing. *Nat. Genet.* 40 1413–1415. 10.1038/ng.259 18978789

[B43] Paz N. Levanon E. Y. Amariglio N. Heimberger A. B. Ram Z. Constantini S. (2007). Altered adenosine-to-inosine RNA editing in human cancer. *Genome Res.* 17 1586–1595. 10.1101/gr.6493107 17908822 PMC2045141

[B44] Paz-Yaacov N. Bazak L. Buchumenski I. Porath H. T. Danan-Gotthold M. Knisbacher B. A. (2015). Elevated RNA editing activity is a major contributor to transcriptomic diversity in tumors. *Cell Rep.* 13 267–276. 10.1016/j.celrep.2015.08.080 26440895

[B45] Peng X. Xu X. Wang Y. Hawke D. H. Yu S. Han L. (2018). A-to-I RNA editing contributes to proteomic diversity in cancer. *Cancer Cell* 33 817–828.e7. 10.1016/j.ccell.2018.03.026 29706454 PMC5953833

[B46] Picardi E. D’Erchia A. M. Gallo A. Pesole G. (2015a). Detection of post-transcriptional RNA editing events. *Methods Mol. Biol.* 1269 189–205. 10.1007/978-1-4939-2291-8_12 25577380

[B47] Picardi E. D’Erchia A. M. Lo Giudice C. Pesole G. (2016). REDIportal: a comprehensive database of A-to-I RNA editing events in humans. *Nucleic Acids Res.* 45 D750–D757. 10.1093/nar/gkw767 27587585 PMC5210607

[B48] Picardi E. Manzari C. Mastropasqua F. Aiello I. D’Erchia A. M. Pesole G. (2015b). Profiling RNA editing in human tissues: towards the inosinome atlas. *Sci. Rep.* 5:14941. 10.1038/srep14941 26449202 PMC4598827

[B49] Picardi E. Pesole G. (2013). REDItools: high-throughput RNA editing detection made easy. *Bioinformatics* 29 1813–1814. 10.1093/bioinformatics/btt287 23742983

[B50] Piechotta M. Wyler E. Ohler U. Landthaler M. Dieterich C. (2017). JACUSA: site-specific identification of RNA editing events from replicate sequencing data. *BMC Bioinformatics* 18:7. 10.1186/s12859-016-1432-8 28049429 PMC5210316

[B51] Pinto Y. Cohen H. Y. Levanon E. Y. (2014). Mammalian conserved ADAR targets comprise only a small fragment of the human editosome. *Genome Biol.* 15:R5. 10.1186/gb-2014-15-1-r5 24393560 PMC4053846

[B52] Porath H. T. Carmi S. Levanon E. Y. (2014). A genome-wide map of hyper-edited RNA reveals numerous new sites. *Nat. Commun.* 5:4726. 10.1038/ncomms5726 25158696 PMC4365171

[B53] Qin Y. R. Qiao J. J. Chan T. H. Zhu Y. H. Li F. F. Liu H. (2014). Adenosine-to-inosine RNA editing mediated by ADARs in esophageal squamous cell carcinoma. *Cancer Res.* 74 840–851. 10.1158/0008-5472.CAN-13-2545 24302582

[B54] Ramaswami G. Li J. B. (2014). RADAR: a rigorously annotated database of A-to-I RNA editing. *Nucleic Acids Res.* 42 D109–D113. 10.1093/nar/gkt996 24163250 PMC3965033

[B55] Riedmann E. M. Schopoff S. Hartner J. C. Jantsch M. F. (2008). Specificity of ADAR-mediated RNA editing in newly identified targets. *RNA* 14 1110–1118. 10.1261/rna.923308 18430892 PMC2390793

[B56] Rosenthal J. J. Seeburg P. H. (2012). A-to-I RNA editing: effects on proteins key to neural excitability. *Neuron* 74, 432–439. 10.1016/j.neuron.2012.04.010 22578495 PMC3724421

[B57] Roth S. H. Danan-Gotthold M. Ben-Izhak M. Rechavi G. Cohen C. J. Louzoun Y. (2018). Increased RNA editing may provide a source for autoantigens in systemic lupus Erythematosus. *Cell Rep.* 23 50–57. 10.1016/j.celrep.2018.03.036 29617672 PMC5905401

[B58] Roth S. H. Levanon E. Y. Eisenberg E. (2019). Genome-wide quantification of ADAR adenosine-to-inosine RNA editing activity. *Nat. Methods* 16 1131–1138. 10.1038/s41592-019-0610-9 31636457

[B59] Roundtree I. A. Evans M. E. Pan T. He C. (2017). Dynamic RNA modifications in gene expression regulation. *Cell* 169 1187–1200. 10.1016/j.cell.2017.05.045 28622506 PMC5657247

[B60] Schwartz S. (2016). Cracking the epitranscriptome. *RNA* 22 169–174. 10.1261/rna.054502.115 26787305 PMC4712667

[B61] Shallev L. Kopel E. Feiglin A. Leichner G. S. Avni D. Sidi Y. (2018). Decreased A-to-I RNA editing as a source of keratinocytes’ dsRNA in psoriasis. *RNA* 24 828–840. 10.1261/rna.064659.117 29592874 PMC5959251

[B62] Silvestris D. A. Picardi E. Cesarini V. Fosso B. Mangraviti N. Massimi L. (2019). Dynamic inosinome profiles reveal novel patient stratification and gender-specific differences in glioblastoma. *Genome Biol.* 20:33. 10.1186/s13059-019-1647-x 30760294 PMC6373152

[B63] Stekhoven D. J. Bühlmann P. (2012). MissForest–non-parametric missing value imputation for mixed-type data. *Bioinformatics* 28 112–118. 10.1093/bioinformatics/btr597 22039212

[B64] Stellos K. Gatsiou A. Stamatelopoulos K. Perisic Matic L. John D. Lunella F. F. (2016). Adenosine-to-inosine RNA editing controls cathepsin S expression in atherosclerosis by enabling HuR-mediated post-transcriptional regulation. *Nat. Med.* 22 1140–1150. 10.1038/nm.4172 27595325

[B65] Tajaddod M. Jantsch M. F. Licht K. (2016). The dynamic epitranscriptome: A to I editing modulates genetic information. *Chromosoma* 125 51–63. 10.1007/s00412-015-0526-9 26148686 PMC4761006

[B66] Tan M. H. Li Q. Shanmugam R. Piskol R. Kohler J. Young A. N. (2017). Dynamic landscape and regulation of RNA editing in mammals. *Nature* 550 249–254. 10.1038/nature24041 29022589 PMC5723435

[B67] Tran S. S. Jun H.-I. Bahn J. H. Azghadi A. Ramaswami G. Van Nostrand E. L. (2019). Widespread RNA editing dysregulation in brains from autistic individuals. *Nat. Neurosci.* 22:25. 10.1038/s41593-018-0287-x 30559470 PMC6375307

[B68] Vlachogiannis N. I. Gatsiou A. Silvestris D. A. Stamatelopoulos K. Tektonidou M. G. Gallo A. (2019). Increased adenosine-to-inosine RNA editing in rheumatoid arthritis. *J. Autoimmun* 106:102329. 10.1016/j.jaut.2019.102329 31493964 PMC7479519

[B69] Zhang L. Yang C.-S. Varelas X. Monti S. (2016). Altered RNA editing in 3′ UTR perturbs microRNA-mediated regulation of oncogenes and tumor-suppressors. *Sci. Rep.* 6:23226. 10.1038/srep23226 26980570 PMC4793219

