# Supplementary material for: Quantifying RNA Editing in Deep Transcriptome Datasets
Source: Front Genet. 2020 Mar 6;11:194. doi: 10.3389/fgene.2020.00194 (PMC7069340; doi:10.3389/fgene.2020.00194)
Supplement: Supplementary file 1 [file Data_Sheet_1.docx]

Supplementary Material

# Supplementary Figures and Tables

## Supplementary Figures


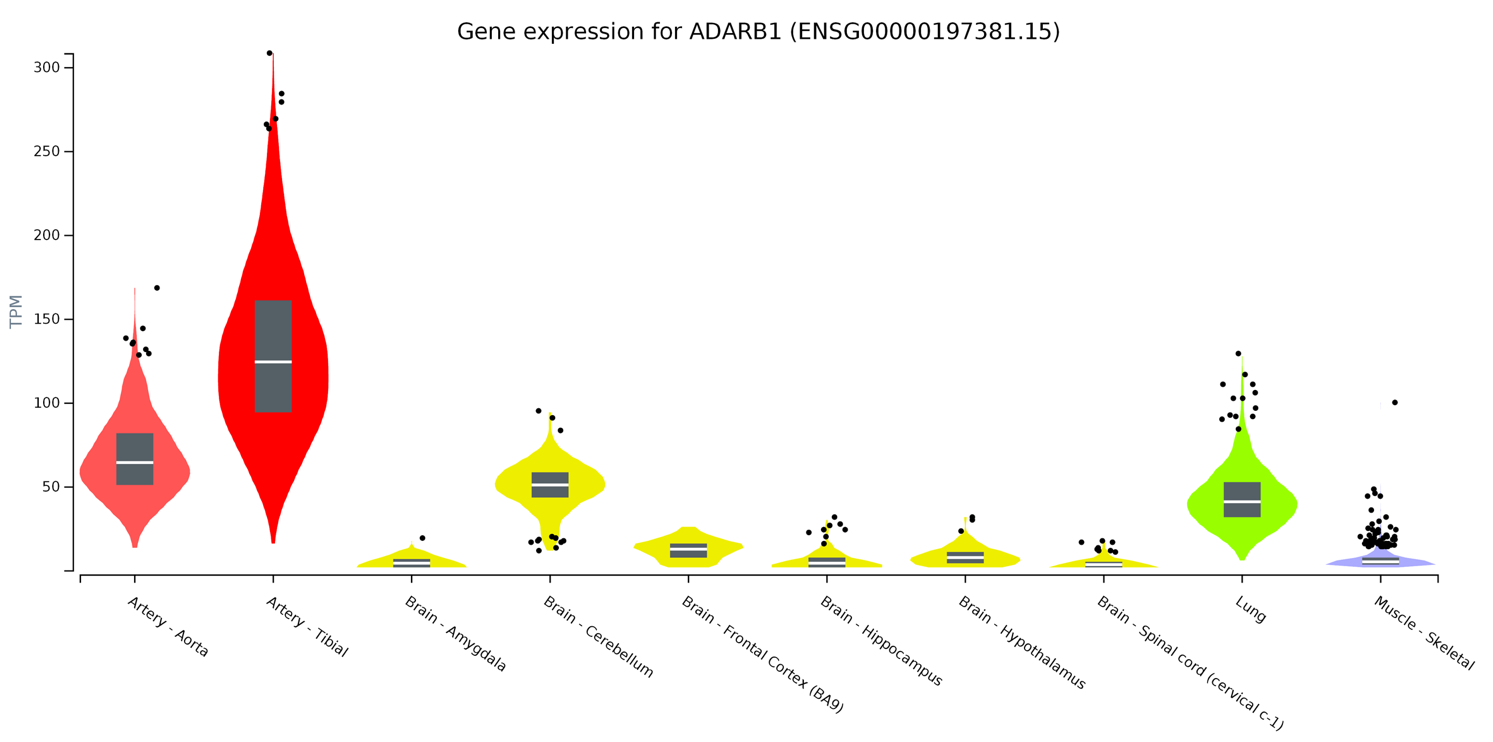


**Supplementary Figure 1.** ADAR2 expression levels from the GTEx Portal.

## Supplementary Tables

**Supplementary Table 1.** List of GTEx RNAseq data used in this work reporting the tissue and “body site” as well as the SRA accession number.

| **Tissue** | **Body Site** | **SRA accession** |
| --- | --- | --- |
| Artery | Aorta | SRR1075579 |
| Artery | Aorta | SRR1083076 |
| Artery | Aorta | SRR1087415 |
| Artery | Aorta | SRR1091254 |
| Artery | Aorta | SRR1101591 |
| Artery | Aorta | SRR1105463 |
| Artery | Aorta | SRR1353028 |
| Artery | Aorta | SRR1368668 |
| Artery | Aorta | SRR1458243 |
| Artery | Aorta | SRR810367 |
| Artery | Aorta | SRR811819 |
| Artery | Aorta | SRR815092 |
| Artery | Aorta | SRR816565 |
| Artery | Aorta | SRR818999 |
| Artery | Tibial | SRR1088437 |
| Artery | Tibial | SRR1099189 |
| Artery | Tibial | SRR612359 |
| Artery | Tibial | SRR612515 |
| Artery | Tibial | SRR612983 |
| Artery | Tibial | SRR613078 |
| Artery | Tibial | SRR613198 |
| Artery | Tibial | SRR613903 |
| Artery | Tibial | SRR615742 |
| Artery | Tibial | SRR661505 |
| Artery | Tibial | SRR662677 |
| Artery | Tibial | SRR663052 |
| Artery | Tibial | SRR810031 |
| Artery | Tibial | SRR814165 |
| Brain | Amygdala | SRR1085015 |
| Brain | Amygdala | SRR1095865 |
| Brain | Amygdala | SRR598671 |
| Brain | Amygdala | SRR599448 |
| Brain | Amygdala | SRR600445 |
| Brain | Amygdala | SRR603534 |
| Brain | Amygdala | SRR607478 |
| Brain | Amygdala | SRR614647 |
| Brain | Amygdala | SRR655435 |
| Brain | Amygdala | SRR657151 |
| Brain | Amygdala | SRR657891 |
| Brain | Amygdala | SRR659625 |
| Brain | Amygdala | SRR661818 |
| Brain | Cerebellum | SRR1093527 |
| Brain | Cerebellum | SRR1398839 |
| Brain | Cerebellum | SRR613747 |
| Brain | Cerebellum | SRR614383 |
| Brain | Cerebellum | SRR657997 |
| Brain | Cerebellum | SRR659331 |
| Brain | Cerebellum | SRR662871 |
| Brain | Cerebellum | SRR663453 |
| Brain | Cerebellum | SRR807657 |
| Brain | Cerebellum | SRR816292 |
| Brain | Cerebellum | SRR817190 |
| Brain | Cerebellum | SRR820468 |
| Brain | Frontal Cortex | SRR1077405 |
| Brain | Frontal Cortex | SRR1084649 |
| Brain | Frontal Cortex | SRR1084842 |
| Brain | Frontal Cortex | SRR1102055 |
| Brain | Frontal Cortex | SRR600361 |
| Brain | Frontal Cortex | SRR602314 |
| Brain | Frontal Cortex | SRR602516 |
| Brain | Frontal Cortex | SRR604262 |
| Brain | Frontal Cortex | SRR604456 |
| Brain | Frontal Cortex | SRR607337 |
| Brain | Frontal Cortex | SRR658307 |
| Brain | Frontal Cortex | SRR661349 |
| Brain | Frontal Cortex | SRR818210 |
| Brain | Hippocampus | SRR1096851 |
| Brain | Hippocampus | SRR1378155 |
| Brain | Hippocampus | SRR1498616 |
| Brain | Hippocampus | SRR607935 |
| Brain | Hippocampus | SRR656564 |
| Brain | Hippocampus | SRR658977 |
| Brain | Hippocampus | SRR660103 |
| Brain | Hippocampus | SRR817751 |
| Brain | Hippocampus | SRR817758 |
| Brain | Hippocampus | SRR817856 |
| Brain | Hippocampus | SRR819134 |
| Brain | Hypothalamus | SRR1071289 |
| Brain | Hypothalamus | SRR1072504 |
| Brain | Hypothalamus | SRR1083100 |
| Brain | Hypothalamus | SRR1389059 |
| Brain | Hypothalamus | SRR598862 |
| Brain | Hypothalamus | SRR602193 |
| Brain | Hypothalamus | SRR604318 |
| Brain | Hypothalamus | SRR608574 |
| Brain | Hypothalamus | SRR613354 |
| Brain | Hypothalamus | SRR660091 |
| Brain | Hypothalamus | SRR661445 |
| Brain | Hypothalamus | SRR661995 |
| Brain | Hypothalamus | SRR665502 |
| Brain | Hypothalamus | SRR820379 |
| Brain | Spinal cord | SRR602598 |
| Brain | Spinal cord | SRR602871 |
| Brain | Spinal cord | SRR612407 |
| Brain | Spinal cord | SRR613807 |
| Brain | Spinal cord | SRR614071 |
| Brain | Spinal cord | SRR615020 |
| Brain | Spinal cord | SRR615731 |
| Brain | Spinal cord | SRR660661 |
| Brain | Spinal cord | SRR661723 |
| Brain | Spinal cord | SRR817880 |
| Lung | Lung | SRR1071568 |
| Lung | Lung | SRR1084602 |
| Lung | Lung | SRR1098998 |
| Lung | Lung | SRR600632 |
| Lung | Lung | SRR612755 |
| Lung | Lung | SRR615129 |
| Lung | Lung | SRR662031 |
| Lung | Lung | SRR808472 |
| Lung | Lung | SRR819186 |
| Muscle | Skeletal | SRR1071955 |
| Muscle | Skeletal | SRR1084417 |
| Muscle | Skeletal | SRR598044 |
| Muscle | Skeletal | SRR612803 |
| Muscle | Skeletal | SRR614852 |
| Muscle | Skeletal | SRR654802 |
| Muscle | Skeletal | SRR656409 |
| Muscle | Skeletal | SRR658989 |
| Muscle | Skeletal | SRR660115 |
| Muscle | Skeletal | SRR660545 |
| Muscle | Skeletal | SRR661639 |
| Muscle | Skeletal | SRR809348 |
| Muscle | Skeletal | SRR815470 |

**Supplementary Table 2.** Number of trimmed and untrimmed reads from human cerebellum sample (accession SRR607967). We also report the number of properly mapped reads (by samtools flagstat) per aligner and genome version (percentages of aligned reads are in brackets).

|  | N. Reads | BWA hg19 | STAR hg19 | HISAT2 hg19 | BWA hg38 | STAR hg38 | HISAT2 hg38 |
| --- | --- | --- | --- | --- | --- | --- | --- |
| Untrimmed | 64901358 | 30969520  (47.72%) | 57876224  (89.17%) | 56336014  (86.80%) | 30968296  (47.71%) | 57518108  (88.62%) | 56751942  (87.44%) |
| Trimmed | 56297926 | 47899340  (85.08%) | 53301850  (94.69%) | 52793196  (93.77%) | 47927744 (85.13%) | 52971976 (94.09%) | 53168512  (94.44%) |
